# The role of high-fiber diets in modulating the gut–lung axis and asthmatic inflammation

**DOI:** 10.3389/fimmu.2026.1825261

**Published:** 2026-07-13

**Authors:** Ke Hua, Fangyuan Hao, Xiaolin Jin, Xiaoyu Li

**Affiliations:** 1 Department of Pediatrics, Chongqing Medical University, Chongqing, China; 2 Laboratory of Innovation, Basic Medical Experimental Teaching Centre, Chongqing Medical University, Chongqing, China

**Keywords:** asthma, gut microbiota, gut-lung axis, high-fiber diet, short-chain fatty acids (SCFAs)

## Abstract

The gut microbiota is a key regulator of human health and disease, exhibiting considerable functional diversity. High-fiber diets are known to profoundly reshape the gut microbial composition and its metabolic output, notably enhancing the production of short-chain fatty acids (SCFAs). These SCFAs mediate communication along the gut–lung axis through multiple mechanisms, including epigenetic modifications, receptor-mediated signaling, and immunomodulation. Consequently, SCFAs promote regulatory T-cell expansion, restore Th1/Th2 balance, and alleviate airway inflammation in asthma. While clinical observations suggest that high-fiber intake can improve asthma symptoms and lung function, its long-term efficacy and generalizability across diverse populations require further investigation. Elucidating the mechanisms of microbial metabolites within the gut–lung axis through multi-omics integration represents a promising option for developing targeted nutritional interventions, particularly for pediatric and treatment-resistant asthma.

## 1 Introduction

In recent years, the gut microbiota has emerged as a critical regulator of host immunity and systemic inflammation (1, 2). Increasing evidence suggests that microbial metabolites act as key mediators linking environmental factors, particularly diet, to immune homeostasis (3). Among these factors, dietary fiber has received considerable attention due to its capacity to reshape gut microbial composition and enhance the production of bioactive metabolites such as short-chain fatty acids (SCFAs), which exert profound immunomodulatory effects (3, 4).

However, despite growing recognition of the role of SCFAs in regulating immune responses—particularly in promoting regulatory T (Treg) cell differentiation—the precise mechanistic pathways by which high-fiber diets influence asthma development through the gut–lung axis remain incompletely understood. In particular, it is still unclear how dietary fiber–induced changes in microbial metabolism translate into systemic immune modulation that directly affects pulmonary inflammation. Moreover, most existing studies focus predominantly on SCFAs, while the potential contributions of other microbiota-derived metabolites, such as bile acids, remain underexplored. This lack of mechanistic integration limits the development of targeted dietary or microbiota-based therapeutic strategies for asthma.

The gut–lung axis, defined as a bidirectional communication network between the gastrointestinal tract and the respiratory system, provides a conceptual framework to understand how gut microbiota-derived signals influence lung immunity (5). Through immune cell trafficking, circulating metabolites, and cytokine signaling, this axis plays a central role in shaping pulmonary immune responses (6). Dysregulation of this axis has been increasingly implicated in the pathogenesis of asthma (7), a chronic inflammatory disease characterized by immune imbalance and airway hyperresponsiveness (8).

In this review, we aim to address these knowledge gaps by systematically examining how high-fiber diets modulate the gut–lung axis through microbiota-derived metabolites. We focus on both classical pathways mediated by SCFAs and emerging regulatory networks involving non-SCFA metabolites, particularly bile acids and their associated signaling pathways. By integrating current evidence on microbial composition, metabolite production, and immune regulation, we seek to provide a comprehensive mechanistic framework linking diet, the gut microbiota, and asthma pathogenesis. Ultimately, this review highlights the potential of microbiota-targeted dietary interventions as a promising strategy for the prevention and management of asthma, especially in pediatric populations.

### 1.1 Regulatory effects of high-fiber diets on gut microbiota composition and function

The heterogeneity of the gut microbiota is shaped by multiple factors including dietary patterns, environmental exposures, and host genetics. Notably, high-fiber diets significantly influence both the varieties and metabolic functionality of gut microbial communities. Dietary fibers exert a profound influence on the gut microbial metabolite profile by serving as fermentable substrates that selectively modulate the composition and metabolic functions of the gut microbiota (9).

Studies have shown that a high-fiber diet significantly influences the composition of the gut microbiota. For instance, inulin, a specific type of high-fiber food, has been found to markedly increase the abundance of *Bifidobacterium* and *Lactobacillus* in the gut. Moreover, different fiber types exhibit distinct microbial responses, pectin enhances butyrate production, whereas resistant starch primarily promotes acetate and propionate formation. These differences may be attributed to the enrichment of specific bacteria: pectin notably increases the abundance of *Prevotella*, while resistant starch stimulates the growth of *Ruminococcus* (10). Furthermore, Lihua Chen et al. corroborated that high-fiber diets considerably increase the abundance of beneficial bacteria including *Lactobacillus*, *Bifidobacterium*, and *Akkermansia* (11, 12), as well as *Firmicutes* (13), *Streptococcus*, *Pseudomonas*, *Lactococcus*, *Acinetobacter*, and *Clostridium* (14, 15), collectively contributing to the maintenance of gut microbiota homeostasis.

A high-fiber diet also promotes the production of beneficial microbial metabolites, including SCFAs such as acetate, propionate, and butyrate (16), as well as bile acids and sphingolipids (17). Furthermore, such a diet modulates the levels of other metabolites, including palmitic acid, stearic acid, and nicotinic acid (18), and can lead to the generation of compounds like trimethylamine N-oxide (TMAO) and indole derivatives (19). Additionally, dietary fiber upregulates genes encoding carbohydrate-active enzymes involved in glycan degradation, thereby enhancing the metabolic activity of the gut microbiota (20). Collectively, these microbial metabolites are pivotal for sustaining anti-inflammatory responses. The effects of different types of dietary fiber on gut microbial composition and function are summarized in **Table 1**.

**Table 1:** Modulation of gut microbiota by different types of dietary fiber.

High-fiber diet type	Microbial taxa	Abundance change	Functional alterations
Inulin	*Bifidobacterium*	+++	↑SCFAs (anti-inflammatory, barrier protection)
	*Lactobacillus*	++	↑Lactate (pH reduction, anti-colonization)
	*Akkermansia*	++	↑Mucin production (barrier restoration)
	*Firmicutes*	++	↑Energy metabolism (butyrate production)
	*Streptococcus*	+	Immunomodulation
	*Pseudomonas*	+	Polysaccharide degradation
	*Lactococcus*	+	↑Lactate (acidification, pathogen inhibition)
	*Clostridium*	+	↑Butyrate production
Pectin	*Prevotella*	+++	↑Butyrate (epithelial protection, anti-inflammatory)
Resistant Starch	*Ruminococcus*	++	↑Acetate/propionate (gluconeogenesis promotion)

### 1.2 Immunomodulatory mechanisms of gut microbiota and metabolites in asthma

Accumulating evidence underscores the critical role of the gut microbiota in the pathogenesis of inflammatory diseases, including asthma, primarily through the modulation of microbial metabolism and host immunity. Epidemiological studies have established a link between early-life gut microbiota profiles and the development of childhood asthma (21). The immunomodulatory role of the gut microbiota extends beyond local intestinal immunity and affects distal pulmonary immune responses via the gut–lung axis. Supporting this concept, a study by Daisky Maruyama et al. demonstrated that a high-fiber diet induces significant alterations in the gut microbial composition and enhances the production of SCFAs, which in turn modulate pulmonary immunity through the gut-lung axis (22). These findings demonstrate that gut microbiota and their metabolites systemically shape immune outcomes via distinct mechanisms, highlighting the central role of microbial communities in immune regulation. 

#### 1.2.1 Immunological basis of bidirectional gut–lung axis communication

**Figure 1:**
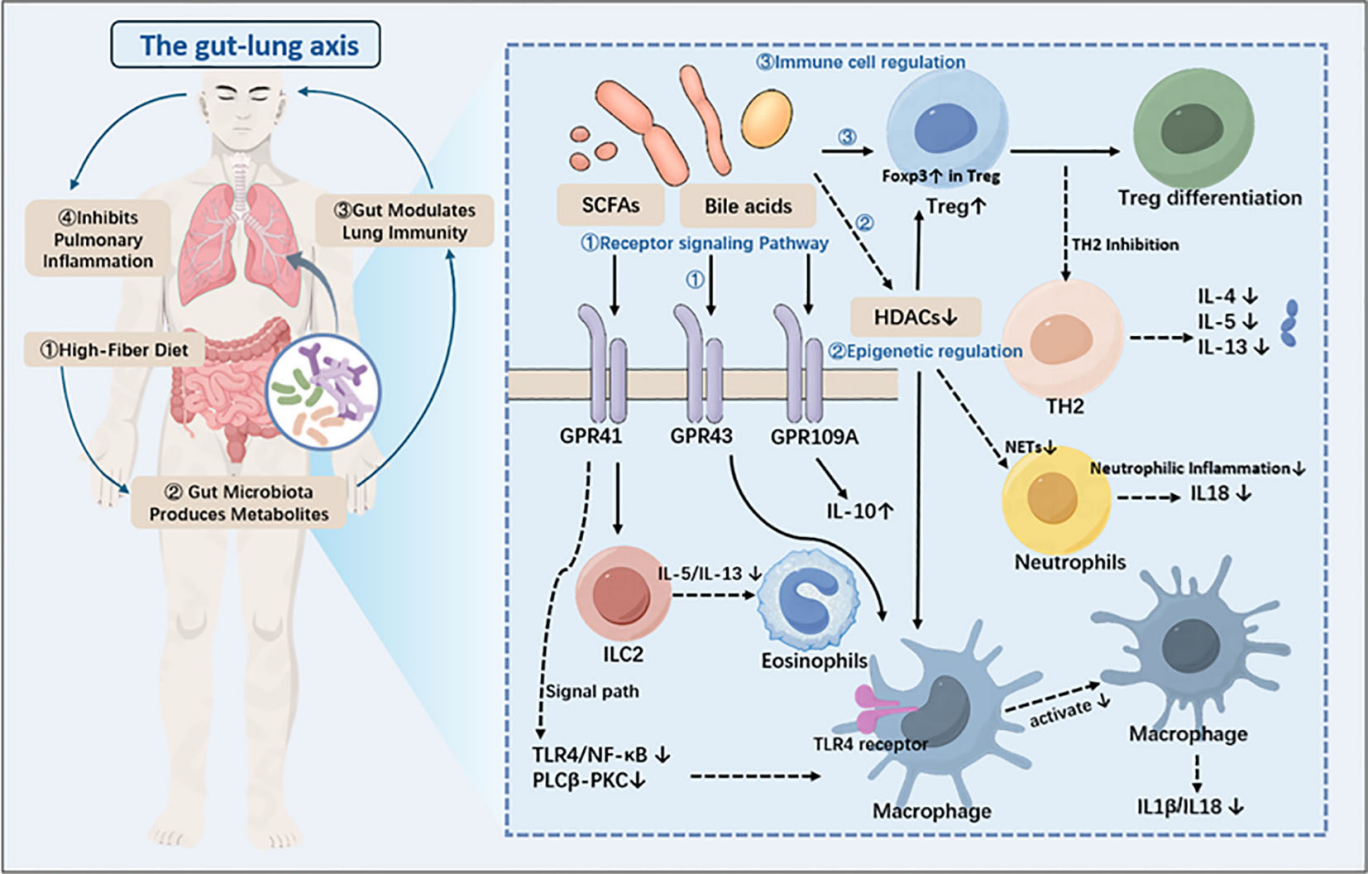
Gut–lung axis: immunomodulatory effects of microbial metabolites. Beneficial gut bacteria ferment dietary fiber to produce key metabolites such as SCFAs and secondary bile acids. These metabolites modulate immune responses through multiple mechanisms: (1) activation of G-protein-coupled receptors (GPR41, GPR43, GPR109A); (2) epigenetic regulation via inhibition of histone deacetylases (HDACs); and (3) direct interaction with immune cells. These actions collectively promote the differentiation and function of Tregs—characterized by high Foxp3 expression—and suppress type 2 immunity by reducing the production of pro-inflammatory cytokines (IL-4, IL-5, IL-13). Additionally, they inhibit the TLR4/NF-κB inflammatory pathway in macrophages, decreasing IL-1β and IL-18 release, and attenuate neutrophil activation and neutrophil extracellular trap (NET) formation, thereby mitigating neutrophilic inflammation. Through the gut–lung axis, these effects systemically dampen excessive immune responses. Solid lines indicate promotional effects; dashed lines indicate inhibitory effects.

The gut–lung axis represents a bidirectional communication network, primarily mediated by the immune system through the trafficking of immune cells, circulation of cytokines, and activation of shared signaling pathways (23). Gut microbiota-derived metabolites exert immunoregulatory effects via systemic circulation. These metabolites not only modulate the function of immune cells, such as T cells (including Treg and Th1/Th2 subsets), dendritic cells (DCs), and type 2 innate lymphoid cells (ILC2s), but also influence cytokine expression and suppress NF-κB signaling activation, thereby attenuating inflammatory responses. The major immunomodulatory mechanisms mediated by microbial metabolites through the gut–lung axis are illustrated in **Figure 1**.

#### 1.2.2 Epigenetic regulation of T cell differentiation by SCFAs

Beyond fixed genetic susceptibility to immune dysregulation, SCFAs represent a malleable layer of regulation, fine-tuning immune signaling pathways through epigenetic mechanisms (24). SCFAs, particularly butyrate and propionate, act as endogenous HDAC inhibitors, primarily targeting HDAC1 and HDAC3 (25). By inhibiting these HDACs, SCFAs promote the hyperacetylation of histone H3 at the promoter and conserved non-coding sequence (CNS) regions of the *Foxp3* locus (26). This modification enhances the transcription of Foxp3, the master regulator of regulatory T cells, thereby directly driving the differentiation and expansion of peripheral Tregs (27).

Concurrently, this HDAC inhibition exerts a suppressive effect on Th2 cell differentiation. Acetylation shifts also downregulate the expression of GATA-3 (the master transcription factor for Th2 cells), leading to a direct reduction in the production of pro-inflammatory Th2 cytokines such as IL-4, IL-5, and IL-13. This dual action—promoting Foxp3 and suppressing GATA-3—serves as a primary epigenetic switch to restore the Treg/Th2 balance in the asthmatic airway (28). Furthermore, butyrate influences the differentiation of other T cell subsets by modulating the expression of additional key transcription factors. Specifically, it upregulates T-bet while downregulating RORs, a transcription factor associated with T helper 17 (Th17) cells, thereby promoting Th1 cell differentiation (29). This shift toward Th1 responses is pivotal for cell-mediated immunity and the clearance of intracellular pathogens, collectively highlighting the indispensable role of microbiota-derived SCFAs in upholding systemic immune homeostasis.

#### 1.2.3 G protein-coupled receptor-mediated immune signaling of SCFAs

SCFAs regulate immune responses through multiple mechanisms, among which activation of G protein-coupled receptors (GPCRs) represents a central pathway. SCFAs bind to receptors such as GPR41 (FFAR3), GPR43 (FFAR2), and GPR109A (HCAR2), triggering distinct yet coordinated intracellular signaling cascades that collectively shape immune cell function and the Treg/Th2 balance (28).

Upon activation, GPR43 couples to both Gi/o and Gq proteins, leading to inhibition of intracellular cAMP accumulation and activation of the PLCβ–PKC and ERK/MAPK pathways (30). These signaling events suppress NF-κB activation in dendritic cells (DCs) and modulate inflammatory cytokine production, thereby limiting the ability of DCs to drive Th2 polarization and promoting a tolerogenic microenvironment conducive to Treg induction (31).

Similarly, GPR41 signals predominantly through Gi/o proteins to reduce cAMP levels and regulate immunometabolic pathways; in the context of allergic inflammation, this signaling influences bone marrow hematopoiesis, leading to the generation of DCs with a tolerogenic phenotype and reduced capacity to initiate Th2-mediated airway inflammation (10).

In contrast, GPR109A, primarily activated by butyrate, signals via Gi-dependent pathways to induce retinaldehyde dehydrogenase (RALDH) expression in macrophages and DCs, while promoting the secretion of anti-inflammatory cytokines such as IL-10 and TGF-β (32). These mediators act on CD4^+^ T cells to enhance Foxp3^+^ Treg differentiation and suppress Th2-associated transcriptional programs, including GATA-3 signaling. In addition to receptor-mediated effects, SCFAs can directly inhibit NF-κB activation, further downregulating pro-inflammatory cytokine expression in innate immune cells (28).

Collectively, the integration of GPCR-mediated signaling pathways, including Gi/o-dependent cAMP suppression, Gq-driven PLCβ–PKC activation, and downstream ERK/MAPK signaling, together with NF-κB inhibition, establishes a coordinated regulatory network that promotes Treg differentiation while restraining Th2-driven inflammation, thereby contributing to immune homeostasis in asthma.

#### 1.2.4 Broad modulation of innate and adaptive immune cells by SCFAs

SCFAs enhance intestinal epithelial tight junctions and barrier function, thereby reducing the translocation of harmful substances and modulating immune cell activity (31). Furthermore, SCFAs regulate inflammatory cells, such as neutrophils, eosinophils, activated macrophages, and type 2 innate lymphoid cells (ILC2s), which are key players in mucosal immunity, contributing to both pathogen defense and the maintenance of immune tolerance. Macrophages play a crucial role in maintaining intestinal immune homeostasis through their dynamic polarization between pro-inflammatory M1 and anti-inflammatory M2 phenotypes (33).Notably, tolerogenic cells, including CD103^+^dendritic cells, under the influence of SCFAs, promote antigen-specific tolerance and help sustain intestinal immune homeostasis (10). Recent work by Sun et al. demonstrated that plant-derived dietary fibers can enhance both microbial butyrate production and intestinal barrier stability, offering a dual mechanism for their systemic protective effects (34).

As a key short-chain fatty acid, butyrate exhibits notable anti-inflammatory properties by promoting the secretion of anti-inflammatory cytokines such as IL-10. More importantly, it regulates the differentiation and function of both Tregs and dendritic cells (DCs). Given that DCs serve as a critical bridge between innate and adaptive immunity, butyrate plays a significant regulatory role in the pathogenesis of allergic asthma through this mechanism (35). By presenting antigen fragments, DCs modulate diverse immune responses, including the activation of T lymphocytes—such as the differentiation of TH1, TH2, and TH17 subsets (36)— and the stimulation of immune responses in ILCs (37).

A study conducted by Daisuke et al, utilizing high-fiber dietary intervention combined with 16S sequencing and metabolomic analysis, revealed that mice fed a high-fiber diet exhibited significant protection against lung injury compared to those on a fiber-free diet (38). This protective effect was closely associated with reduced immune tension in lung tissues, elevated serum propionate levels, and enrichment of specific gut microbiota (39). Further *in vitro* experiments confirmed that propionate attenuates immune responses by inhibiting the secretion of IL-1β and IL-18 from alveolar macrophages, thereby contributing to lung protection (40).

Dysbiosis of the gut microbiota and its diminished metabolic output can compromise immune homeostasis at both local and systemic levels, thereby increasing susceptibility to pulmonary diseases including asthma and viral infections. Allergic asthma is characterized by persistent Th2-polarized immune hyperreactivity triggered by common environmental allergens (41). The core mechanisms involve aberrant activation of multiple immune cells and dysregulation of cytokine networks (42). Specifically, non-pathogenic antigens such as house dust mites and pollen are presented by antigen-presenting cells, leading to the differentiation of CD4^+^ T cells into Th2 cells. These Th2 cells secrete characteristic cytokines including IL-4, IL-5, and IL-13, which promote eosinophil infiltration, IgE production, and airway hyperresponsiveness (AHR), ultimately resulting in chronic airway inflammation and remodeling (43). Notably, SCFAs can counteract this Th2-polarized state by modulating key effector cells, including innate lymphoid type 2 cells (ILC2s), regulatory Tregs, DCs, and eosinophils. For instance, SCFAs directly suppress the production of IL-5 and IL-13 by ILC2s, thereby reducing eosinophil recruitment and AHR (38). Collectively, these mechanisms underscore the crucial role of SCFAs in regulating systemic immunity and their potential to mitigate asthma pathogenesis. The major gut microbiota-derived metabolites and their immunomodulatory mechanisms are summarized in **Table 2**.

**Table 2:** Gut microbiota-derived metabolites and their immunomodulatory networks.

Microbial metabolite	Target cells/pathways	Regulatory mechanisms
SCFAs (Acetate)	GPR43 Receptor	↑ Treg differentiation → ↓ pro-inflammatory cytokine production
SCFAs (Butyrate)	Treg Cells	↑ Histone H3 acetylation → ↓ HDAC activity → ↑ Treg differentiation → ↓ allergic responses
	TH1/TH17 Balance	↑ T-bet expression + ↓ RORγt → ↑ TH1 polarization
	TH2-HDAC11 Pathway	↓ HDAC11 → ↓ IL-4/IL-5 production
	NF-κB Pathway	↓ NF-κB activation → ↓ pro-inflammatory cytokines
	CD103^+^Dendritic Cells	↑ antigen tolerance induction
	Alveolar Macrophages	↓ IL-1β/IL-18 secretion → ↓ pulmonary inflammation
	ILC2 Cells	↓ IL-5/IL-13 production → ↓ eosinophil infiltration
SCFAs (Propionate) Other SCFAs Bile Acids	Peripheral Treg Cells	GPR43/GPR109A activation → ↑ Treg population
	GPR41 Receptor	↓ TLR4-mediated inflammatory responses
	HDAC	Epigenetic regulation → ↓ pro-inflammatory gene expression
	Neutrophils	↓ NET formation/IL-1β release → ↓ neutrophilic inflammation
	Treg cells	↑ Foxp3 expression → ↓ Th2 responses (IL-4/IL-13↓)

#### 1.2.5 Roles of non-SCFAs metabolites (bile acids) in asthma pathogenesis

Beyond SCFAs, gut microbiota-derived metabolites such as succinate, tryptophan derivatives, and secondary bile acids also play significant roles in host metabolism and immune regulation (44). Emerging evidence identifies bile acids (BAs) as complementary immunometabolic regulators. Microbiota-driven conversion of primary bile acids into secondary bile acids (e.g., deoxycholic acid [DCA] and lithocholic acid [LCA]) critically shapes their signaling capacity, whereas dysbiosis-associated reductions in these metabolites are linked to enhanced inflammation and asthma susceptibility (45).

Bile acids regulate airway immunity primarily through three receptor axes. First, the nuclear receptor FXR maintains epithelial homeostasis by enhancing FOXA2 activity, thereby suppressing goblet cell metaplasia and mucus overproduction (46). However, its effects are context-dependent, with potential pro-inflammatory roles under chronic conditions (47). Second, the membrane receptor TGR5 mediates anti-inflammatory responses in innate immunity by activating cAMP–PKA signaling, promoting M2 macrophage polarization, and inhibiting NF-κB and NLRP3 inflammasome activation—pathways particularly relevant to neutrophilic and steroid-resistant asthma (48). Third, LCA-mediated activation of the vitamin D receptor (VDR) suppresses Th2-driven inflammation via induction of Aiolos and inhibition of IL-2/STAT5 signaling, thereby limiting IL-5 and IL-13 production (49).

In addition to these receptor-dependent pathways, conjugated bile acids such as tauroursodeoxycholic acid (TUDCA) exert protective effects by alleviating endoplasmic reticulum stress and reducing epithelial-derived alarmins, representing a complementary non-canonical mechanism (46).

**Figure 2:**
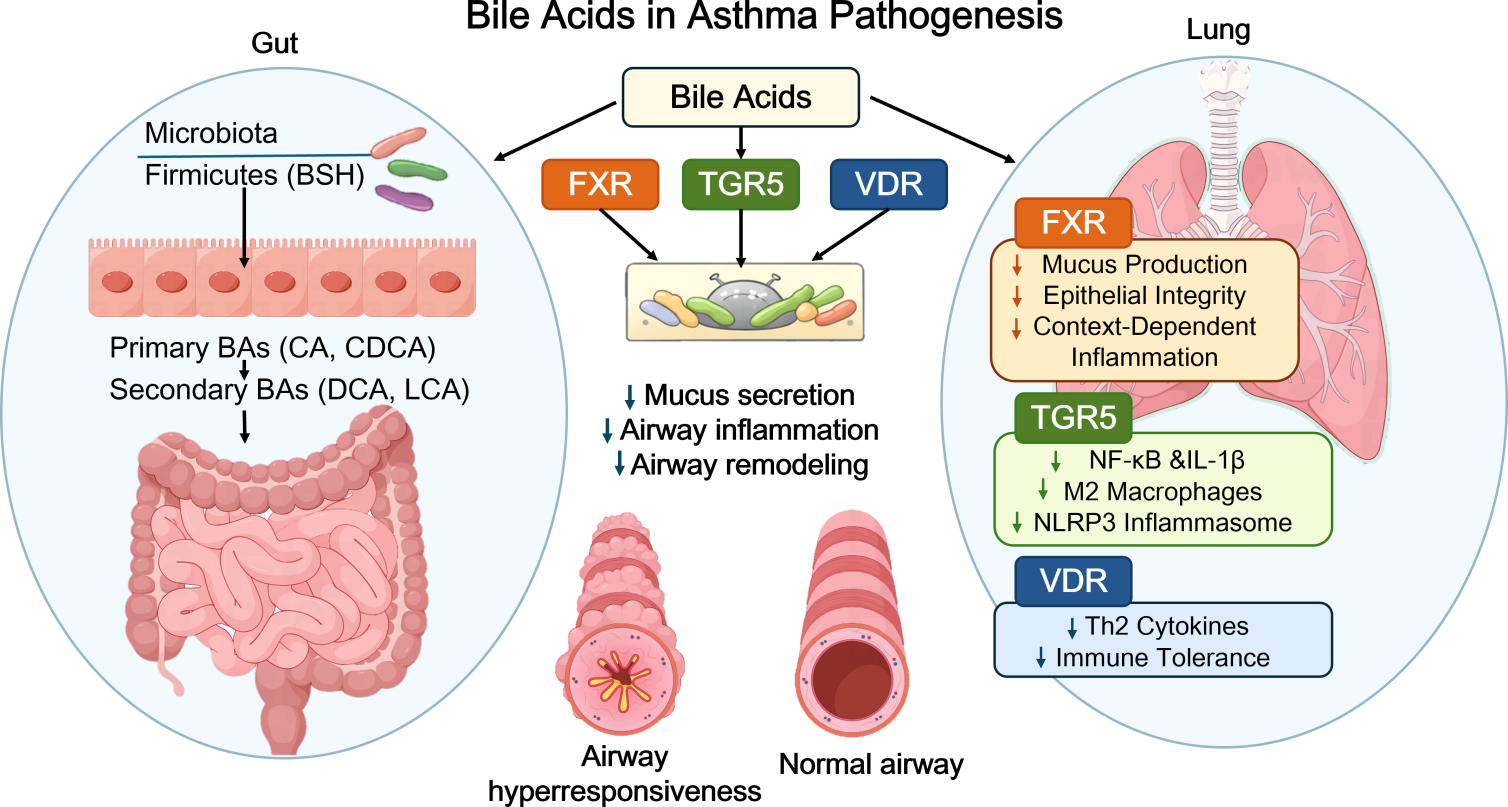
The bile acid signaling network across the gut–lung axis in asthma pathogenesis. Gut microbiota-driven conversion of primary BAs (CA, CDCA) into secondary BAs (DCA, LCA) is critical for immunometabolic regulation. BAs coordinate airway immunity through three distinct receptor axes: (1)FXR Axis: Maintains airway epithelial homeostasis by suppressing mucus production.(2)TGR5 Axis: Mediates anti-inflammatory responses by inhibiting NF-κB and the NLRP3 inflammasome while promoting M2 macrophage polarization (relevant to neutrophilic asthma).(3)VDR Axis: Suppresses adaptive immune responses by reducing Th2 cytokine production. Dysbiosis-associated reductions in secondary BAs disrupt this integrated regulatory network, exacerbating Th2 or neutrophilic inflammation and shifting the airway toward airway hyperresponsiveness.

Collectively, bile acids form an integrated regulatory network across the gut–lung axis, coordinating epithelial integrity (FXR), innate immune suppression (TGR5), and adaptive immune balance (VDR). Disruption of this network shifts immune responses toward Th2 or neutrophilic inflammation, exacerbating asthma pathogenesis. The bile acid signaling network involved in asthma pathogenesis is illustrated in **Figure 2**

### 1.3 Implications of gut microbiota dysbiosis in asthma and clinical perspectives

The homeostasis of the gut microbiota plays a crucial role in modulating host immune balance and influencing susceptibility to disease. Clinical evidence indicates a significant association between gut microbiota dysbiosis and immune-related disorders. For example, children with asthma consistently show a reduced α-diversity of the gut microbiota compared to their healthy counterparts (50). Asthmatic patients also demonstrate marked alterations in microbial composition, characterized by decreased abundance of beneficial genera such as *Bifidobacterium* and *Prevotella*, alongside an increase in potentially pro-inflammatory taxa including *Clostridium* and *Veillonella* (38). These specific structural changes in the gut ecosystem are significantly associated with an increased risk of asthma onset and progression.

In accordance with the PRISMA guidelines, Rabbiya et al. conducted a systematic review and meta-analysis to evaluate the relationship between infant gut microbiota and childhood respiratory diseases. Their findings revealed that a lower relative abundance of *Bifidobacterium* is closely associated with asthma or atopic wheezing in children aged 1 to 7 years (51).

**Figure 3:**
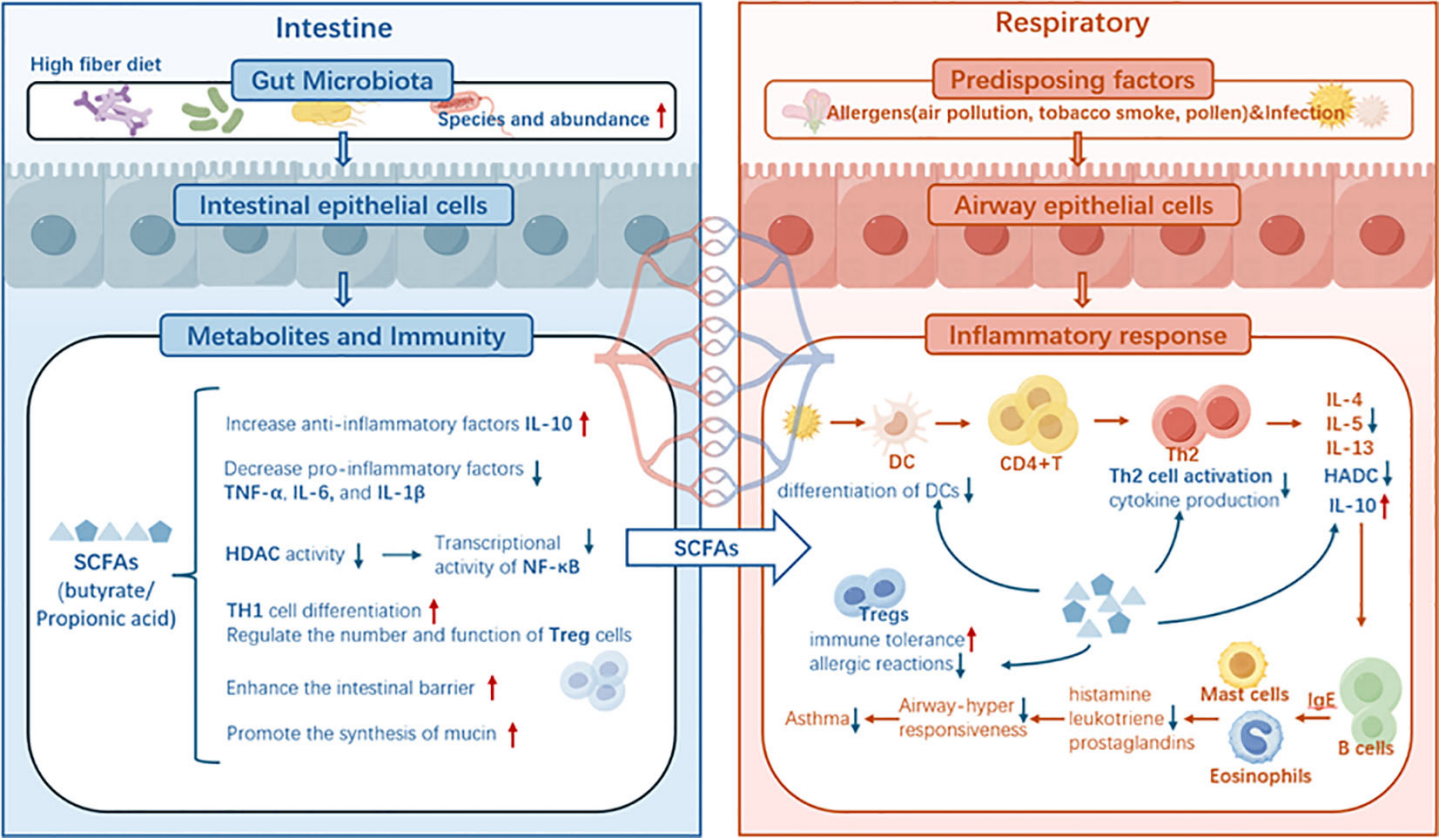
High-fiber diet modulates the gut microbiota–immune axis to alleviate asthmatic inflammation. A high-fiber diet enhances the diversity and abundance of the gut microbiota. The major microbial metabolites—SCFAs contribute significantly to immune regulation by: - Increasing the expression of the anti-inflammatory cytokine IL-10; - Decreasing the expression of pro-inflammatory cytokines such as TNF-α, IL-6, and IL-1β; - Inhibiting histone deacetylase (HDAC) activity and suppressing NF-κB signaling, thereby reducing inflammatory responses; - Promoting the differentiation of Tregs, enhancing immune tolerance, and attenuating allergic reactions. SCFAs, along with modulated cytokines and immune cells, can reach the lungs via systemic circulation. There, they alleviate asthma symptoms by inhibiting Th2 cell activation and the production of associated cytokines, ultimately reducing airway inflammation.

Consistent with these results, children diagnosed with asthma or atopic wheezing consistently showed significantly reduced levels of *Bifidobacterium*. As a dominant genus in the infant gut, *Bifidobacterium* has been demonstrated to play a key role in immune regulation and development—promoting the maturation of gut-associated lymphoid tissue and enhancing the generation of Tregs (52). Moreover, *Bifidobacterium* produces surface-exposed exopolysaccharides that can modulate host B-cell responses and attenuate excessive immune activation. A schematic overview of the mechanisms by which a high-fiber diet modulates the gut microbiota–immune axis to alleviate asthmatic inflammation is presented in **Figure 3**.

## 2 Discussion

This review synthesizes key recent evidence on the mechanisms by which high-fiber diets modulate the gut microbiota and shape inflammatory responses in asthma. Dietary fiber intake critically influences the composition and metabolic function of the gut microbiota. It notably enriches beneficial genera such as Bifidobacterium and Lactobacillus and stimulates the production of SCFAs. These effects collectively contribute to the maintenance of intestinal homeostasis. 

Gut microbiota-derived metabolites play a central role in regulating immune and inflammatory responses. Among these metabolites, SCFAs serve as key mediators in the gut–lung axis. SCFAs exert systemic anti-inflammatory effects through three primary mechanisms: (i) activation of GPCRs, (ii) epigenetic regulation via HDAC inhibition, and (iii) direct modulation of immune cell differentiation and function. In addition, secondary bile acids—such as lithocholic acid (LCA) and deoxycholic acid (DCA)—contribute to asthma pathogenesis by interacting with signaling pathways involving FXR, TGR5, and VDR. Together, these metabolites fine-tune immune balance and help maintain intestinal and respiratory homeostasis.

The stability of the gut microbiota is therefore essential for systemic immune regulation, and its dysbiosis is strongly implicated in asthma development (53). Clinical evidence indicates that children with asthma exhibit reduced gut microbial α-diversity, characterized by a decline in beneficial taxa such as *Bifidobacterium* and an expansion of pro-inflammatory genera—a profile consistently associated with increased disease risk.

Although current research provides valuable insights into the effects of high-fiber diets on the gut–lung axis and asthmatic inflammation, a more comprehensive understanding of the underlying mechanisms and therapeutic potential is needed to bridge existing knowledge gaps.

### 2.1 Limitations in metabolite research

Current studies have primarily focused on the immunomodulatory roles of SCFAs—such as butyrate, propionate, and acetate. However, the signaling mechanisms through which SCFAs act via G protein-coupled receptors and HDACs are not yet fully understood, particularly their specific targets and downstream pathways in immune cells (54). Moreover, the potential contributions of other microbiota-derived metabolites to asthma pathogenesis are poorly characterized. Although high-fiber diets are known to influence the production of metabolites like TMAO, indole derivatives, and bile acids, their precise functions and mechanisms in asthma-related inflammation require further clarification.

A systematic understanding of the broader metabolic network in asthma is still lacking. Future studies should integrate transcriptomic (e.g., RNA-seq) and proteomic (e.g., mass spectrometry) approaches to elucidate how these metabolites affect gene expression and protein profiles in immune cells. This will help construct an integrated regulatory network linking metabolites, genes, and proteins in asthma.

In addition to current metabolite-centered approaches, emerging research highlights the importance of microbial ecological networks and cross-kingdom interactions, particularly the interplay between bacteria and fungi (the gut mycobiome), in shaping host immune responses. Although bacterial communities have been extensively studied, fungal components of the microbiota remain underexplored in the context of asthma. Recent evidence suggests that fungi may interact with bacterial populations to influence metabolic outputs and immune signaling, thereby contributing to the regulation of inflammatory responses along the gut–lung axis.

Furthermore, advances in meta-omics technologies, including metagenomics, metatranscriptomics, metabolomics, and integrated multi-omics analyses, provide powerful tools to dissect these complex interactions at a systems level. By enabling simultaneous characterization of microbial composition, functional gene expression, and metabolite profiles, these approaches can facilitate the construction of comprehensive microbiota–metabolite–immune networks. Future studies integrating cross-kingdom microbiome analysis with multi-omics data will be essential to uncover novel regulatory mechanisms and identify potential therapeutic targets in asthma.

### 2.2 Discrepancies between animal models and human studies

Current understanding of the gut-lung axis in asthma relies heavily on findings from animal studies, whose translational relevance to human asthma needs further validation (55). Most clinical trials conducted so far have been limited by short intervention durations and small sample sizes, which reduces their statistical power and limits conclusions regarding long-term efficacy and safety. Furthermore, the lack of large-scale randomized controlled trials (RCTs) and comprehensive meta-analyses represents a significant evidence gap, limiting the ability to draw definitive conclusions about the efficacy of dietary interventions.

While animal models (e.g., OVA-induced asthmatic mice) are indispensable for elucidating molecular mechanisms such as GPCR signaling, significant translational discrepancies exist between these models and human clinical manifestations. Firstly, laboratory animals are maintained in highly standardized SPF environments, lacking the complex “exposome” encountered by humans—including air pollution, diverse allergens, and early-life antibiotic exposure—which significantly shift the immune system’s baseline state. Secondly, inherent differences in intestinal anatomy and microbiota composition (e.g., the *Firmicutes* to *Bacteroidetes* ratio) between rodents and humans mean that identical fiber interventions may yield markedly different SCFA profiles across species. Furthermore, human asthma is characterized by high heterogeneity (diverse endotypes), whereas single animal models often fail to replicate the interference of common clinical comorbidities, such as obesity and insulin resistance, on treatment responses.

To bridge the existing translational gap, future research should prioritize large-scale, long-term randomized controlled trials (RCTs) that focus on establishing standardized dose-response relationships for structurally diverse fibers across specific asthma phenotypes. By extending intervention periods to over six months and integrating multi-omics—such as metagenomics and systemic metabolomics—researchers can transcend routine clinical monitoring to validate the mechanistic causal chain between microbial metabolites and immune homeostasis. Furthermore, stratified studies targeting high-risk subpopulations, including early childhood cohorts for immune priming and adult obese or steroid-resistant asthmatics for metabolic modulation, are essential to advancing precision nutritional interventions in clinical practice (56).

### 2.3 The incompletely defined role of microbiota diversity in asthma

The nature of the association between gut microbial composition/function and asthma pathogenesis has not been definitively established. While some studies report reduced microbial diversity in asthma patients, others show no significant differences, underscoring that a causal link remains to be fully characterized (57). The functional impact of specific bacterial taxa is also debated, as their roles may vary considerably depending on host genetics, diet, and environmental exposures (58). This heterogeneity highlights the need for study designs that adequately account for these confounding factors.

To address these gaps, large-scale cohort and case-control studies are needed, incorporating multi-omics approaches such as metagenomics and metatranscriptomics to deeply characterize microbial diversity and functional profiles in relation to asthma phenotypes (59). In parallel, mechanistic studies using *in vitro* cell cultures and animal models should investigate how metabolites derived from specific bacteria (e.g., *Bifidobacterium, Bacteroides*) modulate immune cell function. Coupled with clinical validation in well-defined patient cohorts, these efforts may help identify candidate microbial targets for precision interventions in asthma.

### 2.4.Clinical translation and future perspectives

A clinical trial conducted by Nottingham Trent University (ClinicalTrials.gov ID: NCT02872675) enrolled 17 participants to investigate the effects of the prebiotic HOST-DM059 on gut bacterial metabolites and systemic inflammatory markers in adults with and without respiratory allergy-induced bronchospasm. After four weeks of prebiotic supplementation, significant improvements in lung function were observed in asthmatic adults compared to the placebo group. However, the small sample size warrants caution in interpreting these preliminary findings.

Two additional ongoing clinical trials are further evaluating the effects of high-fiber diets and pre/probiotics on asthma outcomes. One is a three-month randomized controlled trial (ClinicalTrials.gov ID: NCT06005506) involving 64 adult asthma patients, assessing the impact of combined dietary fiber and probiotic supplementation on SCFA levels, lung function parameters (FEV_1_ and FVC), and Asthma Control Test scores. While this sample size represents an improvement over previous studies, larger-scale validation remains necessary.Another 12-week randomized controlled trial (ClinicalTrials.gov ID: NCT05949255) is investigating daily probiotic intake in 20 obese asthmatic patients with insulin resistance. This study aims to evaluate changes in the metabolome (including SCFAs and lactate), asthma biomarkers (FeNO, IgE, blood cell counts, inflammatory cytokines), and clinical outcomes (lung function FEV_1_ /FVC, ACT, and AQLQ scores). The limited cohort size highlights the need for future expanded trials.

These studies underscore the need for more refined and individualized approaches in future research. Precision nutrition represents a promising direction for asthma management. Variations in baseline gut microbiota composition, metabolic status (e.g., obesity and insulin resistance), asthma phenotype, and disease severity may lead to differential responses to nutritional interventions. Moreover, fundamental differences exist between pediatric and adult asthma: childhood asthma is closely linked to early immune and microbiome development (60), whereas adult asthma often involves established airway remodeling and metabolic comorbidities. Thus, clinical application of high-fiber diets should be tailored to specific populations. For children, dietary strategies should focus on guiding microbial colonization and shaping long-term immune tolerance. In adults, interventions should emphasize metabolic modulation—leveraging SCFAs to ameliorate inflammation, insulin resistance, and potentially enhance corticosteroid sensitivity. Furthermore, microbiota-targeted therapies may offer adjunct treatment options for patients with steroid-resistant asthma, who respond poorly to conventional inhaled corticosteroids (ICS), by modulating immune-inflammatory pathways (61).

Future studies should therefore seek to integrate multi-omics data with clinical characteristics to identify biomarkers predictive of treatment response. This will enable the design of tailored microbiota-targeted interventions—such as specific probiotic strains, prebiotics, or synbiotics—to maximize clinical benefits for distinct asthma subpopulations.

## 3 Conclusion

A high-fiber diet significantly alters the composition and metabolic function of the gut microbiota, enhances the abundance of beneficial bacterial species, and promotes the production of metabolites such as SCFAs. Through multiple mechanisms, including immunomodulation and the gut–lung axis, these changes contribute to the reduction of airway inflammation and amelioration of asthma symptoms. Although current research provides valuable insights into how high-fiber diets modulate asthmatic inflammation via the gut microbiota, further in-depth studies and clinical validation are necessary to resolve existing controversies and limitations. Future efforts should focus on developing personalized intervention strategies targeting the gut microbiota, with the ultimate goal of improving the health and quality of life of asthma patients.

## References

[1] Wagenaar CA van de Put M Bisschops M Walrabenstein W de Jonge CS Herrema H . The effect of dietary interventions on chronic inflammatory diseases in relation to the microbiome: a systematic review. Nutrients. (2021) 13:3208. doi: 10.3390/nu13093208 34579085 PMC8464906

[2] Xu X Zhang F Ren J Zhang H Jing C Wei M . Dietary intervention improves metabolic levels in patients with type 2 diabetes through the gut microbiota: a systematic review and meta-analysis. Front Nutr. (2023) 10:1243095. doi: 10.3389/fnut.2023.1243095 38260058 PMC10800606

[3] Ross FC Patangia D Grimaud G Lavelle A Dempsey EM Ross RP . The interplay between diet and the gut microbiome: implications for health and disease. Nat Rev Microbiol. (2024) 22:671–686. doi: 10.1038/s41579-024-01068-4 39009882

[4] Williams EJ Berthon BS Stoodley I Williams LM Wood LG . Nutrition in asthma. Semin Respir Crit Care Med. (2022) 43:646–661. doi: 10.1055/s-0042-1742385 35272384

[5] Leduc L Costa M Leclère M . The microbiota and equine asthma: an integrative view of the gut-lung axis. Animals (Basel). (2024) 14:253. doi: 10.3390/ani14020253 38254421 PMC10812655

[6] Chunxi L Haiyue L Yanxia L Jianbing P Jin S . The gut microbiota and respiratory diseases: new evidence. J Immunol Res. (2020) 2020:2340670. doi: 10.1155/2020/2340670 32802893 PMC7415116

[7] Su M Tang T Tang W Long Y Wang L Liu M . Astragalus improves intestinal barrier function and immunity by acting on intestinal microbiota to treat T2DM: a research review. Front Immunol. (2023) 14:1243834. doi: 10.3389/fimmu.2023.1243834 37638043 PMC10450032

[8] Zhou Y Li Q Pan R Wang Q Zhu X Yuan C . Regulatory roles of three miRNAs on allergen mRNA expression in Tyrophagus putrescentiae. Allergy. (2022) 77:469–482. doi: 10.1111/all.15111 34570913

[9] Yang A Ye Y Liu Q Xu J Li R Xu M . Response of nutritional values and gut microbiomes to dietary intake of ω-3 polyunsaturated fatty acids in Tenebrio molitor larvae. Insects. (2025) 16:970. doi: 10.3390/insects16090970 41009149 PMC12471062

[10] Tan JK Macia L Mackay CR . Dietary fiber and SCFAs in the regulation of mucosal immunity. J Allergy Clin Immunol. (2023) 151:361–370. doi: 10.1016/j.jaci.2022.11.007 36543697

[11] Chen L Liu B Ren L Du H Fei C Qian C . High-fiber diet ameliorates gut microbiota, serum metabolism and emotional mood in type 2 diabetes patients. Front Cell Infect Microbiol. (2023) 13:1069954. doi: 10.3389/fcimb.2023.1069954 36794003 PMC9922700

[12] Wang X Xu T Liu R Wu G Gu L Zhang Y . High-fiber diet or combined with acarbose alleviates heterogeneous phenotypes of polycystic ovary syndrome by regulating gut microbiota. Front Endocrinol (Lausanne). (2021) 12:806331. doi: 10.3389/fendo.2021.806331 35185786 PMC8847200

[13] Huang Z Boekhorst J Fogliano V Capuano E Wells JM . Impact of high-fiber or high-protein diet on the capacity of human gut microbiota to produce tryptophan catabolites. J Agric Food Chem. (2023) 71:6956–6966. doi: 10.1021/acs.jafc.2c08953 PMC10176579 37126824

[14] Jin L Huang Y Yang S Wu D Li C Deng W . Diet, habitat environment and lifestyle conversion affect the gut microbiomes of giant pandas. Sci Total Environ. (2021) 770:145316. doi: 10.1016/j.scitotenv.2021.145316 33517011

[15] Zhang W Jia T Zhang H Zhu W . Effects of high-fiber food on gut microbiology and energy metabolism in Eothenomys miletus at different altitudes. Front Microbiol. (2023) 14:1264109. doi: 10.3389/fmicb.2023.1264109 37727288 PMC10505965

[16] Perler BK Friedman ES Wu GD . The role of the gut microbiota in the relationship between diet and human health. Annu Rev Physiol. (2023) 85:449–468. doi: 10.1146/annurev-physiol-031522-092054 36375468

[17] Jang YO Kim OH Kim SJ Lee SH Yun S Lim SE . High-fiber diets attenuate emphysema development via modulation of gut microbiota and metabolism. Sci Rep. (2021) 11:7008. doi: 10.1038/s41598-021-86404-x 33772084 PMC7997879

[18] Zhong Y Cao J Ma Y Zhang Y Liu J Wang H . Fecal microbiota transplantation donor and dietary fiber intervention collectively contribute to gut health in a mouse model. Front Immunol. (2022) 13:842669. doi: 10.3389/fimmu.2022.842669 35185934 PMC8852624

[19] Palombaro M Raoul P Cintoni M Rinninella E Pulcini G Aspromonte N . Impact of diet on gut microbiota composition and microbiota-associated functions in heart failure: a systematic review of in vivo animal studies. Metabolites. (2022) 12:1271. doi: 10.3390/metabo12121271 36557307 PMC9787978

[20] Wastyk HC Fragiadakis GK Perelman D Dahan D Merrill BD Yu FB . Gut-microbiota-targeted diets modulate human immune status. Cell. (2021) 184:4137–4153.e14. doi: 10.1016/j.cell.2021.06.019 PMC9020749 34256014

[21] Li R Guo Q Zhao J Kang W Lu R Long Z . Assessing causal relationships between gut microbiota and asthma: evidence from two sample Mendelian randomization analysis. Front Immunol. (2023) 14:1148684. doi: 10.3389/fimmu.2023.1148684 37539057 PMC10394653

[22] Liu Q Tian X Maruyama D Arjomandi M Prakash A . Lung immune tone via gut-lung axis: gut-derived LPS and short-chain fatty acids' immunometabolic regulation of lung IL-1β, FFAR2, and FFAR3 expression. Am J Physiol Lung Cell Mol Physiol. (2021) 321:L65–L78. doi: 10.1152/ajplung.00421.2020 PMC8321849 33851870

[23] Song XL Liang J Lin SZ Xie YW Ke CH Ao D . Gut-lung axis and asthma: a historical review on mechanism and future perspective. Clin Transl Allergy. (2024) 14:e12356. doi: 10.1002/clt2.12356 38687096 PMC11060082

[24] Chen E Chen C Chen F Yu P Lin L . Positive association between MIC gene polymorphism and tuberculosis in Chinese population. Immunol Lett. (2019) 213:62–69. doi: 10.1016/j.imlet.2019.07.008 31400356

[25] He J Zhang P Shen L Niu L Tan Y Chen L . Short-chain fatty acids and their association with signalling pathways in inflammation, glucose and lipid metabolism. Int J Mol Sci. (2020) 21:6356. doi: 10.3390/ijms21176356 32887215 PMC7503625

[26] Guo Y Shi J Wang Q Hong L Chen M Liu S . Metformin alleviates allergic airway inflammation and increases Treg cells in obese asthma. J Cell Mol Med. (2021) 25:2279–2284. doi: 10.1111/jcmm.16269 PMC7882927 33421348

[27] Wang N Li C Zhang Z . Arctigenin ameliorates high-fat diet-induced metabolic disorders by reshaping gut microbiota and modulating GPR/HDAC3 and TLR4/NF-κB pathways. Phytomedicine. (2024) 135:156123. doi: 10.1016/j.phymed.2024.156123 39396403

[28] Mann ER Lam YK Uhlig HH . Short-chain fatty acids: linking diet, the microbiome and immunity. Nat Rev Immunol. (2024) 24:577–595. doi: 10.1038/s41577-024-01014-8 38565643

[29] Gaskarth DA Fan S Highton AJ Kemp RA . The microbial metabolite butyrate enhances the effector and memory functions of murine CD8+ T cells and improves anti-tumor activity. Front Med (Lausanne). (2025) 12:1577906. doi: 10.3389/fmed.2025.1577906 40630475 PMC12234554

[30] Maslowski KM Vieira AT Ng A Kranich J Sierro F Yu D . Regulation of inflammatory responses by gut microbiota and chemoattractant receptor GPR43. Nature. (2009) 461:1282–1286. doi: 10.1038/nature08530 PMC3256734 19865172

[31] Zong Y Wang X Wang J . Research progress on the correlation between gut microbiota and preeclampsia: microbiome changes, mechanisms and treatments. Front Cell Infect Microbiol. (2023) 13:1256940. doi: 10.3389/fcimb.2023.1256940 38029244 PMC10644267

[32] Lee MJ Park YM Kim B Tae IH Kim NE Pranata M . Disordered development of gut microbiome interferes with the establishment of the gut ecosystem during early childhood with atopic dermatitis. Gut Microbes. (2022) 14:2068366. doi: 10.1080/19490976.2022.2068366 35485368 PMC9067516

[33] Wu S Fang X Zhao J Liu G Liao P . Nutrient regulation targeting macrophage-controlled intestinal mucosal healing: a promising strategy against intestinal mucositis induced by deoxynivalenol. Toxicon. (2025) 264:108434. doi: 10.1016/j.toxicon.2025.108434 40451542

[34] Sun W Jia J Liu G Liang S Huang Y Xin M . Polysaccharides extracted from old stalks of Asparagus officinalis L. improve nonalcoholic fatty liver by increasing the gut butyric acid content and improving gut barrier function. J Agric Food Chem. (2025) 73:6632–6645. doi: 10.1021/acs.jafc.4c07078 40042965

[35] Varricchi G Ferri S Pepys J Poto R Spadaro G Nappi E . Biologics and airway remodeling in severe asthma. Allergy. (2022) 77:3538–3552. doi: 10.1111/all.15473 PMC10087445 35950646

[36] Schreiber S Hammers CM Kaasch AJ Schraven B Dudeck A Kahlfuss S . Metabolic interdependency of Th2 cell-mediated type 2 immunity and the tumor microenvironment. Front Immunol. (2021) 12:632581. doi: 10.3389/fimmu.2021.632581 34135885 PMC8201396

[37] Aghapour M Ubags ND Bruder D Hiemstra PS Sidhaye V Rezaee F . Role of air pollutants in airway epithelial barrier dysfunction in asthma and COPD. Eur Respir Rev. (2022) 31:220112. doi: 10.1183/16000617.0112-2021 PMC9128841 35321933

[38] Zhao X Hu M Zhou H Yang Y Shen S You Y . The role of gut microbiome in the complex relationship between respiratory tract infection and asthma. Front Microbiol. (2023) 14:1219942. doi: 10.3389/fmicb.2023.1219942 37577440 PMC10413575

[39] Shen Q Chen J Yang S Zhang H Yu H Wang S . Protection against cigarette smoke-induced chronic obstructive pulmonary disease via activation of the SIRT1/FoxO1 axis by targeting microRNA-132. Am J Transl Res. (2024) 16:5516–5524. doi: 10.62347/fvqp4019 PMC11558385 39544778

[40] Maruyama D Liao WI Tian X Bredon M Knapp J Tat C . Regulation of lung immune tone by the gut-lung axis via dietary fiber, gut microbiota, and short-chain fatty acids. bioRxiv. (2023). doi: 10.1101/2023.08.24.552964

[41] Wu Y Zeng M Jiao X Ma X Wang H Chen Y . Protective effects of pollenin B in asthma: PPAR-γ-mediated regulation of inflammatory pathways and arachidonic acid metabolism. Phytomedicine. (2025) 145:156975. doi: 10.1016/j.phymed.2025.156975 40544731

[42] Hongyan L . Esculetin attenuates Th2 and Th17 responses in an ovalbumin-induced asthmatic mouse model. Inflammation. (2016) 39:735–743. doi: 10.1007/s10753-015-0300-4 26797918

[43] Barrios RJ Kheradmand F Batts L Corry DB . Asthma: pathology and pathophysiology. Arch Pathol Lab Med. (2006) 130:447–451. doi: 10.5858/2006-130-447-apap 16594736

[44] Guo P Zeng M Liu M Zhang Y Jia J Zhang Z . Zingibroside R1 isolated from Achyranthes bidentata Blume ameliorates LPS/D-GalN-induced liver injury by regulating succinic acid metabolism via the gut microbiota. Phytother Res. (2025) 39:4520–4534. doi: 10.1002/ptr.70067 40836368

[45] Tong Y Lou X . Interplay between bile acids, gut microbiota, and the tumor immune microenvironment: mechanistic insights and therapeutic strategies. Front Immunol. (2025) 16:1638352. doi: 10.3389/fimmu.2025.1638352 40821794 PMC12354525

[46] Yu Y Geng S Bu C Cao G Han Y Xie D . Dry ginger and Schisandra chinensis modulate intestinal flora and bile acid metabolism to treatment asthma. Front Microbiol. (2025) 16:1541335. doi: 10.3389/fmicb.2025.1541335 40212383 PMC11984949

[47] Campbell C Kandalgaonkar MR Golonka RM Yeoh BS Vijay-Kumar M Saha P . Crosstalk between gut microbiota and host immunity: impact on inflammation and immunotherapy. Biomedicines. (2023) 11:294. doi: 10.3390/biomedicines11020294 36830830 PMC9953403

[48] Tan JK McKenzie C Mariño E Macia L Mackay CR . Metabolite-sensing G protein-coupled receptors-facilitators of diet-related immune regulation. Annu Rev Immunol. (2017) 35:371–402. doi: 10.1146/annurev-immunol-051116-052235 28446062

[49] Losol P Wolska M Wypych TP Yao L O'Mahony L Sokolowska M . A cross talk between microbial metabolites and host immunity: its relevance for allergic diseases. Clin Transl Allergy. (2024) 14:e12339. doi: 10.1002/clt2.12339 38342758 PMC10859320

[50] Rao D Meyer J Maurer M Shiyanbola OO . Erratum to "Perceptions of psychosocial and interpersonal factors affecting self-management behaviors among African Americans with diabetes" [Exploratory Research in Clinical and Social Pharmacy 3C (2021) 100057. Explor Res Clin Soc Pharm. (2022) 8:100196. doi: 10.1016/j.rcsop.2022.100196 36304940 PMC9593260

[51] Aslam R Herrles L Aoun R Pioskowik A Pietrzyk A . Link between gut microbiota dysbiosis and childhood asthma: Insights from a systematic review. J Allergy Clin Immunol Glob. (2024) 3:100289. doi: 10.1016/j.jacig.2024.100289 39105129 PMC11298874

[52] Alcazar CG Paes VM Shao Y Oesser C Miltz A Lawley TD . The association between early-life gut microbiota and childhood respiratory diseases: a systematic review. Lancet Microbe. (2022) 3:e867-e880. doi: 10.1016/s2666-5247(22)00184-7 35988549 PMC10499762

[53] Zhang Y Deng J Chen T Liu S Tang Y Zhao JR . Formononetin alleviates no reflow after myocardial ischemia-reperfusion via modulation of gut microbiota to inhibit inflammation. Life Sci. (2024) 358:123110. doi: 10.1016/j.lfs.2024.123110 39374772

[54] Milani C Duranti S Bottacini F Casey E Turroni F Mahony J . The first microbial colonizers of the human gut: Composition, activities, and health implications of the infant gut microbiota. Microbiol Mol Biol Rev. (2017) 81. doi: 10.1128/mmbr.00036-17 29118049 PMC5706746

[55] Salameh M Burney Z Mhaimeed N Laswi I Yousri NA Bendriss G . The role of gut microbiota in atopic asthma and allergy, implications in the understanding of disease pathogenesis. Scand J Immunol. (2020) 91:e12855. doi: 10.1111/sji.12855 31793015

[56] Liu Y Zhou Y Zhang H Zhao K Yang D . Gut-lung axis mediates asthma pathogenesis: Roles of dietary patterns and their impact on the gut microbiota. Exp Mol Pathol. (2025) 142:104964. doi: 10.1016/j.yexmp.2025.104964 40194490

[57] Barcik W Boutin RCT Sokolowska M Finlay BB . The role of lung and gut microbiota in the pathology of asthma. Immunity. (2020) 52:241–55. doi: 10.1016/j.immuni.2020.01.007 32075727 PMC7128389

[58] Saeed NK Al-Beltagi M Bediwy AS El-Sawaf Y Toema O . Gut microbiota in various childhood disorders: Implication and indications. World J Gastroenterol. (2022) 28:1875–901. doi: 10.3748/wjg.v28.i18.1875 35664966 PMC9150060

[59] Jovandaric MZ Jovanović K Raus M Babic S Igic T Kotlica B . The significance of plant nutrition in the creation of the intestinal microbiota-prevention of chronic diseases: A narrative review. Med (Kaunas). (2024) 60. doi: 10.3390/medicina60121969 39768848 PMC11678629

[60] Tang D Wang C Liu H Wu J Tan L Liu S . Asthma in children: A brief review for primary care providers. Pediatr Ann. (2019) 48:e103-e109. doi: 10.3928/19382359-20190219-01 30874817

[61] Tang D . Integrated multi-omics analysis reveals mountain-cultivated ginseng ameliorates cold-stimulated steroid-resistant asthma by regulating interactions among microbiota, genes, and metabolites. Int J Mol Sci. (2024) 25. doi: 10.3390/ijms25169110 39201796 PMC11354367

